# Knot tying in arthroplasty and arthroscopy causes lesions to surgical gloves: a potential risk of infection

**DOI:** 10.1007/s00167-022-07136-7

**Published:** 2022-09-01

**Authors:** Andreas Enz, Annett Klinder, Lucas Bisping, Christoph Lutter, Philipp Warnke, Thomas Tischer, Wolfram Mittelmeier, Robert Lenz

**Affiliations:** 1 Orthopaedic Clinic and Policlinic, University Medicine Rostock, Doberanerstraße 142, 18057 Rostock, Germany; 2 Institute of Medical Microbiology, Virology and Hygiene, University Medicine Rostock, Rostock, Germany

**Keywords:** Surgical site infection, Glove damage, EN455, Microperforation, Arthroscopy, Arthroplasty

## Abstract

**Purpose:**

Recent studies have shown that the incidence of glove lesions during arthroscopy is much lower than that during primary and revision arthroplasty. However, the rate of glove damage after knot tying has not yet been systematically recorded. Therefore, the aim of the study was to determine the impact of surgical knot tying on glove integrity. It was hypothesized that knot tying increases the rate of glove damage, especially in arthroscopic surgery, which could be of special relevance in the treatment of rotator cuff tears.

**Methods:**

Gloves that were changed immediately before suturing and only worn during knot tying were investigated for their integrity by means of water tightening test according to EN455. A total of 234 gloves from 40 total hip arthroplasties (THAs), 42 total knee arthroplasties (TKAs) and 36 rotator cuff repairs (RCRs) were collected. A bacterial pass-through test (BPTT) on glove lesions was performed under simulated sterile surgical conditions for 3 surgeons after a wear duration of 45 min.

**Results:**

Glove damage by knot tying occurred in 25% of THA, 36.6% of TKA and 25% of RCR surgeries. In THA, the pulling hand (PH) was affected in 46.2%, and the main area of damage (15.4%) was detected on the tip of the middle finger; in TKAs the PH was damaged in 75%, and in RCRs the PH was affected in 66.7%, with most of the lesions (20% each) occurring on the tip of the index finger and the ring finger. The BPTT showed *Staphylococcus hominis and Bacillus cereus.*

**Conclusion:**

Intraoperative knot tying causes damage to gloves, which is of special relevance for arthroscopic surgery. Whereas knot tying is only partly responsible for glove damage in arthroplasty, the general rate of glove damage in arthroscopic surgery is low without knot tying. The surgical knot tying process must be understood as a possible damaging impact on the glove. Therefore, single gloving is not recommended, which is especially important in arthroscopic surgery, where double gloving is not yet standard.

**Level of Evidence:**

IV.

**Supplementary Information:**

The online version contains supplementary material available at 10.1007/s00167-022-07136-7.

## Introduction

The prevention of infections during surgical procedures is of great importance, with the hand of the surgeon being one possible source of pathogens. In addition to surgical hand disinfection, the surgical glove is one of the most important cornerstones in the prevention of infections for the patient and the surgical team [16]. Recent studies have shown high rates of glove damage in joint and revision arthroplasty, procedures where gloves are subjected to repetitive mechanical stress during surgery. As a result, the integrity of the gloves is compromised [4, 10, 11, 33]. However, glove damage was also described in previous studies during much less mechanically demanding operations, such as joint arthroscopies and laparoscopic procedures [9–11, 22].

It is conceivable that particularly larger damage to the gloves can occur in major orthopaedic surgeries, such as hip and knee or revision arthroplasty, due to mechanical stress and the instruments used, whereas in low-impact operations, such as shoulder arthroscopy, the lesion rate is lower, and the majority of lesions are one millimetre or less (microlesions) in size [10, 11]. However, the causes of these lesions have not yet been clarified. Whether procedures such as tying fascia during hip or knee arthroplasty or the technique in the treatment of rotator cuff lesions have any effect has not yet been systematically investigated in the literature. The influence of knot tying has mainly been described under laboratory settings [14, 23]. Intraoperative studies under real surgical conditions are rare; in particular, the influence of intraoperative surgical knot tying on sterile gloves in minimally invasive shoulder surgery is poorly understood [20]. To our knowledge, this is the first study to measure the extent of damage to gloves by intraoperative knot tying.

As surgical gloves are inexpensive single-use items, their importance is commonly underestimated. Many surgeons rely on standardized regulations (EN 455, ASTM D3577) regarding infection and self-protection. It goes largely unnoticed that they are production rather than protection standards. Intraoperative mechanical stress is not represented in any of these standards. EN 455 defines holes as a defect in the glove that allows water to leak out. For the water tightening test, a glove is clamped in a holding device and filled with 1000 ml of water (temperature = 15–35 °C); if no water leaks out within 2–3 min, the glove is considered leak-proof. A production lot must meet a leak tightness level of an acceptable quality limit (AQL) of 0.65. Mechanistically, only the tensile force of the entire glove is tested, which must be above 9 newtons (1 *N* = weight of a bar of chocolate) [13].

A coherent study of the influence of knot tying on glove damage in the three major joints (shoulder, hip, knee) has not yet been available in the literature. In particular, differences in the localization and size of glove damage among the entities have not been analysed. Differences in damage configuration between penetrating and friction lesions have not been documented thus far. For surgeons, knowledge of the different susceptibilities to glove damage depending on the joint is extremely important for preventing complications. However, glove lesions could play an important role in both self-protection and patient safety. The hypothesis is that the damage due to knot tying in the shoulder, hip and knee interventions differs in localization and size depending on the type of surgery.

## Materials and methods

At the orthopaedic clinic and policlinic, gloves from 40 total hip arthroplasty (THA), 41 total knee arthroplasty (TKA) and 36 arthroscopic rotator cuff repairs (RCR) were collected from March until August 2020. From these interventions, 234 gloves were subsequently tested with water tightening test according to the European standard EN 455. Ethics approval for the study was granted by the local ethics committee (A2016-0112), and data protection requirements were observed.

The suture material for the closure of the fascia/capsule after hip and knee joint replacement was Vicryl 2 (Ethicon, Inc., Johnson & Johnson Intl., NJ, USA). The sutures on the rotator cuff were stitched with Fibrewire 2 (Arthrex, Inc., FL, USA). THA and TKA were sutured with two concurrent knots presented as slip knots, the fascia was closed in tension, and an opposing knot was tied under tension conditions. This was followed up by two further safety knots. In RCR, seven knots were tied arthroscopically as a standard of secure treatment. The pulling thread was passed through a knot pusher, and the end of the thread was looped several times around the ring finger of the pulling hand. Two identical knots were tied with the looping hand as slip knots and pushed to the end of the thread with the knot pusher, followed by a reverse locking knot. Further safety knots were tied in 2 to 1 alternations for a total 7 knots.

**Fig. 1:**
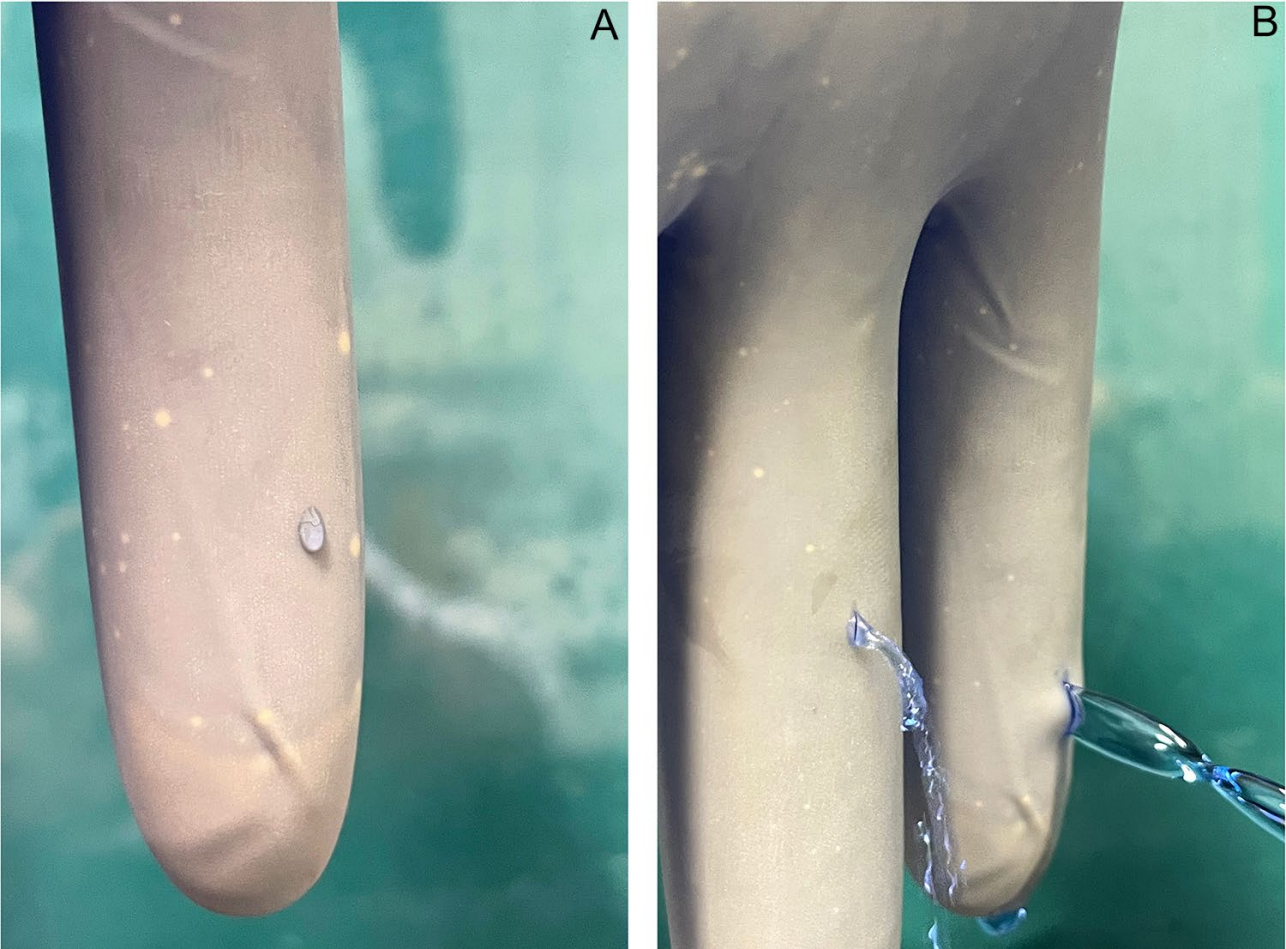
Illustration of glove lesions during the water tigtening test in ascending size. Figure A shows the typical drop formation of a microlesion (< 1 mm). Figure B shows the water leakage from a 2 mm lesion (left finger shown) and a 4 mm lesion (right finger shown). The test fluid was stained with blue ink for illustrative purposes

The gloves of the knot-tying surgeons were collected and packed at the end of each surgery, and the data relevant for evaluation were documented. The total number of gloves, number of gloves per operation, and type and duration of the operation were recorded. A separate pair of gloves was worn solely for the knot tying procedure (tendon, capsule, and fascia) to exclude damage from the previous steps of the surgery. All surgeries were performed with sterile, powder-free latex gloves for single use (ProtexisTM, Cardinal Health Dublin, Ohio, USA (AQL 0.65). The examination of the gloves was conducted at the Biomechanics and Implant Technology Research Laboratory of the Orthopaedic Clinic and Policlinic. To determine the baseline of holes during production, 100 unused gloves from the manufacturer were tested. As a control, the influence of glove undressing was tested on 50 gloves without surgery. The evaluation was performed according to standard EN 455, “Medical gloves for single use—Part 1”, a method for testing for freedom from holes with the water tightening test (Fig. 1). The localization of the damage was determined, the size and dimension were measured with a plastic goniometer (Kirchner & Wilhelm GmbH & Co. KG, Asperg, Germany) with an accuracy of ± 1 mm, and the lesion configuration was recorded microscopically (Digital Microscope VHX-6000, Keyence, Germany).

To analyse the possible passage of bacteria through a glove lesion, simulated surgical conditions without a patient were set up. Three surgeons, who were dressed in a sterile gown and one pair of sterile gloves, disinfected their hands with Sterillium (PAUL HARTMANN AG, Heidenheim, Germany) for 3 min according to EN 12,791 [12]. This was followed by the handling of surgical instruments (hammer, clamps, scissors, luers, etc.) in the operating room under lamina air flow conditions for 45 min. After 22 min into the wear duration (45 min), the fingertips of the gloves were intentionally damaged palmar with a solid surgical spring-eyed needle (HS54, OESHS54, RESORBA Medical GmbH, Nuremberg, Germany). Each fingertip was punctured with a separate sterile needle. Instrument use was performed for an additional 13 min, followed by 10 min of free manual and arthroscopic knot tying with knot sliding instruments. Subsequently, each finger was pressed onto an aerobic and anaerobic agar plate (COLUMBIA 5% SB and SCHAEDLER 5% SB, Becton Dickinson GmbH, Heidelberg, Germany) for 4 s under sterile conditions. The plates were microbiologically examined in the in-house Institute for Microbiology, Virology and Hygiene according to German microbiological standards at a microbiological laboratory accredited by the national accreditation organisation of the Federal Republic of Germany (DAkkS) DIN EN ISO 15189 and DIN EN ISO/IEC 17,025. A total of 120 samples were taken and evaluated. Among them, 60 samples were in the “glove lesion” group, with 30 aerobic and 30 anaerobic samples. In the “gloveless” group, 60 samples were taken, including 30 aerobic and 30 anaerobic samples. The agar plates were incubated for a total of 5 days. As a control, each surgeon performed the same procedure under the same setup without gloves the next day (comparison group).

### Statistical analysis

The collected data were analysed using SPSS Statistics Package Version 22 (IBM Corp., New York, USA). Descriptive statistics were calculated for continuous and categorical variables. Continuous variables are displayed as the mean values and standard deviations (SD) as well as the median and range, as most of the data were not normally distributed. Categorical factors are shown as frequencies (*n*) with percentages in brackets. Testing for differences between different types of operations of categorical factors was performed by Pearson's chi-square test. The significance level was set at *p* < 0.05. Sample size calculation was based on analysis with ANOVA (Cohen`s *f* = 0.2; alpha level 0.05; power 0.7). A total of 198 test items were calculated, with 66 test items per intervention group, resulting in 33 surgical procedures.

## Results

### Overview data and examined surgical treatment

In THA and TKA, knot tying procedures were performed by a total of 10 endoprosthetic surgeons, while three shoulder specialists performed knot tying in the rotator cuff interventions as well as in THA and TKA. In the arthroplasties, seven tying surgeons used the left hand to pull the thread and knotted over the thread with the right hand, and six knotters used the right hand to pull the thread. In RCR, two of the three shoulder surgeons used the knot pusher in the right hand, looping the ring finger with the pulling thread several times and then tying the right hand over the thread. One surgeon used the knot pusher in the left hand, but the knot tying technique itself was identical. All surgeons were right-handed.

### Glove handling

In the determination of the baseline, no prior damage to the 100 tested gloves was detected. The glove batch was within the AQL of 0.65. Of the 50 gloves that were put on and taken off, none showed damage.

### Number of glove lesions, localization on the glove and size

**Fig. 2:**
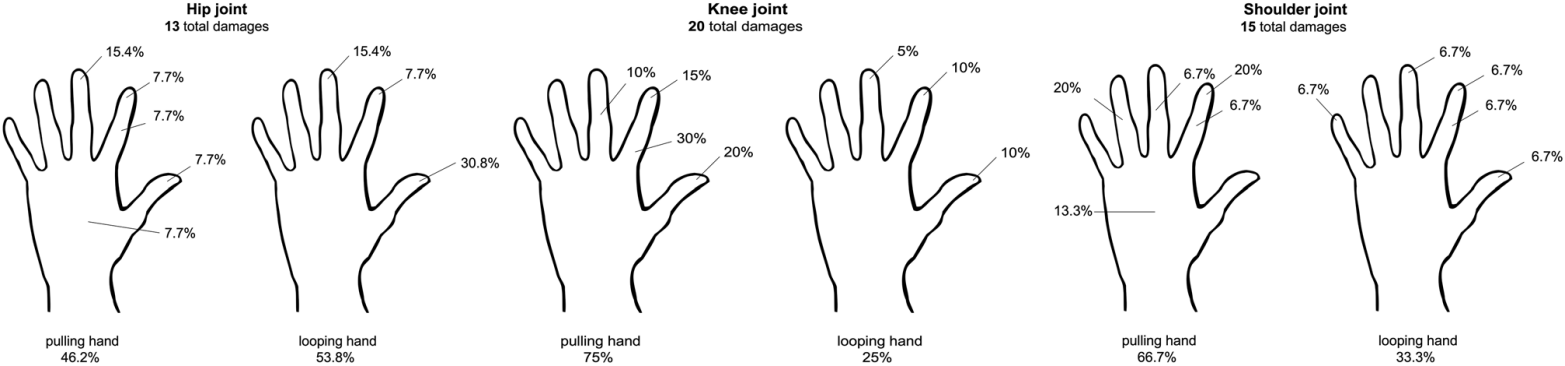
Representation of the percentage damage distribution of the lesions over the affected gloves according to the individual operations. Data in %

**Fig. 3:**
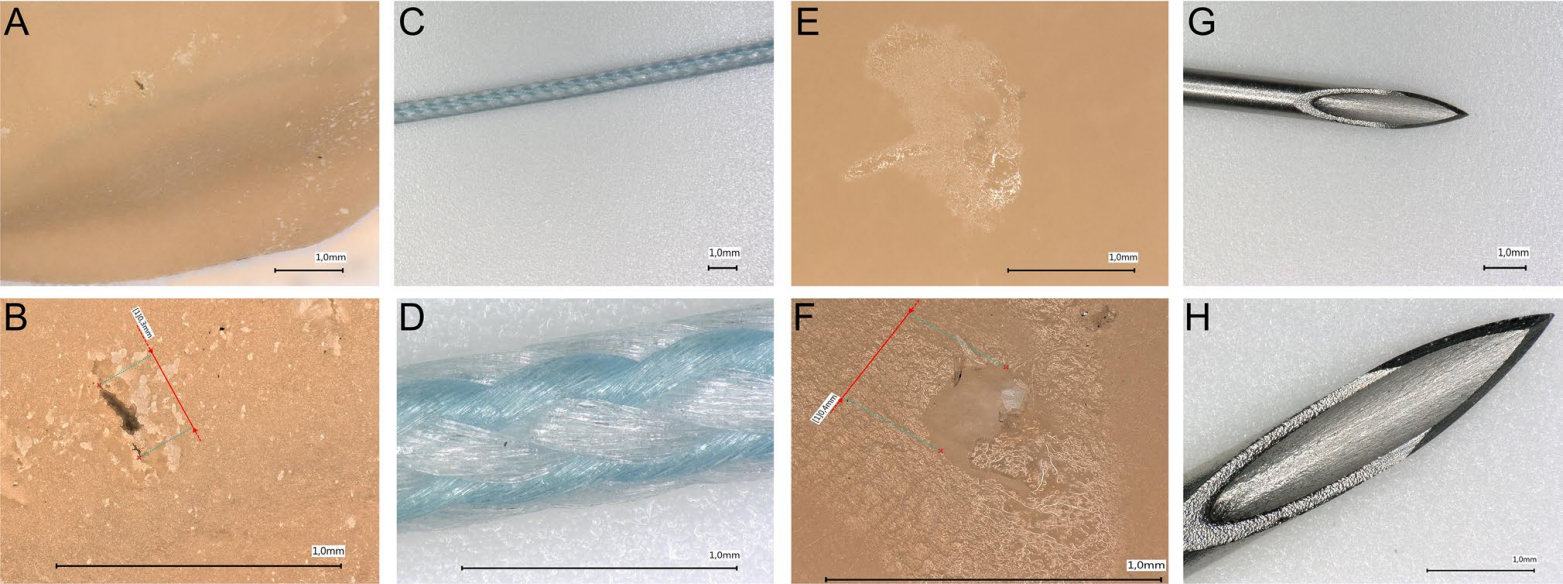
Images A and B show typical friction lesions with tear formation in the glove after the use of nonabsorbable suture material for suturing tendons (**A** in 20 × magnification, **B** in 200 × magnification). **C** and **D** show the Fiberwire suture material used (**C** in 20 × magnification, **B** *n* 200 × magnification). **E** and **F** show a hole caused by a 20G cannula (**E** at 20 × magnification, **F** at 200 × magnification). **G** and **H** show a typical cannula used during arthroscopy (20G 0.9 × 2 3/4)

In total, 234 gloves were collected, and 48 instances of damage were detected. Detailed information is provided in Tables 1 and 2, as well as in Fig. 2. Penetrating and friction injuries showed different injury patterns. Figure 3 shows a typical knot tying and penetrating lesion.

**Table 1:** Descriptive analysis of surgery and patient data

Patient/operation data	Hip	Knee	Rotator cuff	Total	*P* value
Body mass index [M ± SD; MD (range)	27.3 (± 4.5)	33.1 (± 5.7)	27.9 (± 4.7)	29.5 (± 5.6)	
Male	21	13	26	60	
Female	19	28	10	57	
Number of operations	40	41	36	117	
Total number of gloves used (*n*)	80	82	72	234	
Damaged [*n*, (%)]	12 (15.0%)	16 (19.5%)	11 (15.2%)	39 (16.7%)	
+PH	5 (46.2%)	11 (75.0%)	6 (66.7%)	23 (58.9%)	0.237^#^
LH	7 (53.8%)	5 (25.0%)	5 (33.3%)	16 (41.1%)	0.788^#^
Undamaged [*n*, (%)]	68 (85.0%)	66 (80.5%)	61 (84.8%)	197 (83.3%)	
Damages in total (*n*)	13	20	15	48	
PH	6^a^ (46.0%)	15^b^ (75.0%)	10^b^ (67.0%)	31	< 0.001^#^
LH	7^a^ (54.0%)	5^b^ (25.0%)	5^b^ (33.0%)	17	
Number of operations					
With damaged gloves [*n*, (%)]	10 (25.0%)	15 (36.6%)	9 (25.0%)	34 (29.1%)	0.420^#^
Without damaged gloves [*n*, (%)]	30 (75.0%)	26 (63.4%)	27 (75.0%)	83 (70.9%)	

^#^ Pearson-Chi-Quadrat, ^a,b^ = same small letters show no significance, different letters show significance, *SD* standard deviation, *M* mean, *MD* median, *PH* pulling hand, *LH* looping hand

**Table 2:** Representation of the distribution of intraoperative damage and its size distribution according to the surgical procedure performed (numbers bold, percent in brackets)

Total number of damages Localizations	Hips		Knee		Rotator cuff		*P* value
	13		20		15		< 0.001*
	PH	LH	PH	LH	PH	LH	
Thumb	(0.0)	0.0	0.0	0.0	0.0	0.0	
Tip of thumb	1 (7.7)	4 (30.8)	4 (20.0)	2 (10.0)	0.0	1 (6.7)	
Index finger	1 (7.7)	0.0	6 (30.0)	0.0	1 (6.7)	0.0	
Tip of index finger	1 (7.7)	1 (7.7)	3 (15.0)	2 (10.0)	3 (20.0)	1 (6.7)	
Index phalanx	0.0	0.0	2 (10.0)	0.0	0.0	1 (6.7)	
Middle finger	0.0	0.0	0.0	0.0	1 (6.7)	0.0	
Tip of middle finger	2 (15.4)	2 (15.4)	0.0	1 (5.0)	0.0	1 (6.7)	
Ring finger	0.0	0.0	0.0	0.0	3 (20.0)	0.0	
Tip of ring finger	0.0	0.0	0.0	0.0	0.0	0.0	
Little finger	0.0	0.0	0.0	0.0	0.0	0.0	
Tip of little finger	0.0	0.0	0.0	0.0	0.0	1 (6.7)	
Palm	0.0	0.0	0.0	0.0	2 (13.3)	0.0	
Dorsal FA/back of the hand	1 (7.7)	0.0	0.0	0.0	0.0	0.0	
Palm FA	0.0	0.0	0.0	0.0	0.0	0.0	
Thumb/index finger	0.0	0.0	0.0	0.0	0.0	0.0	
Index finger/middle finger	0.0	0.0	0.0	0.0	0.0	0.0	
Percent per PH/LH	6 (46.2)	7 (53.8)	15 (75.0)	5 (25.0)	10 (66.7)	5 (33.3)	

Sizes of damage (mm) / Data in %	PH	LH	PH	LH	PH	LH	*P* value n.s.*
1 mm	4 (30.8)	7 (53.8)	15 (75.0)	3 (15.0)	8 (53.3)	4 (26.7)	
2 mm	0.0	0.0	0.0	1 (5.0)	2 (13.3)	0.0	
3 mm	0.0	0.0	0.0	0.0	0.0	0.0	
4 mm	0.0	0.0	0.0	1 (5.0)	0.0	0.0	
5 mm	0.0	0.0	0.0	0.0	0.0	0.0	
6 mm	0.0	0.0	0.0	0.0	0.0	0.0	
7 mm	0.0	0.0	0.0	0.0	0.0	1 (6.7)	
8 mm	0.0	0.0	0.0	0.0	0.0	0.0	
9 mm	0.0	0.0	0.0	0.0	0.0	0.0	
10 mm	1 (7.7)	0.0	0.0	0.0	0.0	0.0	
> 10 mm	1 (7.7)	0.0	0.0	0.0	0.0	0.0	

^*^Pearson-Chi-Quadrat was performed over all locations as well as sizes, *n.s*  not significant, *PH* pulling hand, *LH* looping hand, *FA* forearm

### Bacterial pass-through testing

Evaluations occurred on Days 1, 2 and 5 (anaerobic only on Day 5). In the samples of the "glove lesion" group, *S. hominis* was detected for Surgeon 2 on the left index finger (1 colony-forming unit (CFU) anaerobic) and Surgeon 3 on the right small finger (1 aerobic CFU and 1 anaerobic CFU). In the "gloveless" group, *B. cereus* was detected for Surgeon 1 (thumb: 6 aerobic CFUs and 9 anaerobic CFUs, index finger: 1 aerobic CFUs/3 anaerobic CFUs, middle finger: 8 aerobic CFUs/5 anaerobic CFUs, ring finger: 10 aerobic CFUs/7 anaerobic CFUs, little finger: 8 aerobic CFUs/5 anaerobic CFUs) and Surgeon 2 (index finger: 1 anaerobic CFU, ring finger: 1 anaerobic CFU). In the anaerobic approach, no obligate anaerobes were found.

## Discussion

The most important findings of our study were as follows: (1) Knot tying is a potential cause of glove damage in RCRs, THAs, and TKAs. (2) The localization of glove damage varies between joints and types of intervention. (3) Lesion size was found to be predominantly less than 1 mm (microlesions). (4) Microlesions allow bacterial passage, thus providing a potential intraoperative source of contamination, especially in single gloving.

To date, there is limited information on the exact origin of the damage and when it occurs in the time course of an intervention [26].

The development of microlesions and the influence of surgical knots on glove microlesions have not been conclusively clarified [14, 23]. The process of knot tying appears to permanently damage the integrity of the glove. As shown in this study, surgical knot tying primarily caused microlesions. Thus, some of the lesions on gloves described in the literature could be explained, even during mechanically less demanding procedures such as soft tissue surgery [9, 24, 32]. As shown, surgical hand disinfection can provide an effect of germ reduction over a period of 45 min, but longer-duration surgeries could result in recurrence of pathogen formation, as alcohol-based disinfectants have a rapid germicidal effect on the skin but no lasting residual activity [27, 28]. As the recovery of bacterial skin flora after the use of hand disinfection is slow, glove lesions could play a role in prolonged operations or in the later course of the surgery. [15].

Because the knot tying process occurs mainly at the end of an operation, microlesions could play a special role, as skin flora forms again in the course of time; thus, the risk of contamination of the sterile joint cavities could occur. The size of the lesion is only of minor importance here because pathogens with a size of less than 1.0 µm are considerably smaller than the damages found and can thus penetrate through them easily. The friction and traction force created by the suture at the interface between skin and glove and suture and glove as well as other forces could therefore lead to contamination if this occurs in a lesion area [23]. In particular, the highest damage rate on the pulling index finger was found across different clinical disciplines and types of surgery, and this could play a role in surgical site infections in this context.

While double gloving was performed in this study, double gloving should be a widely used standard, and it is not consistently used in other clinical disciplines [3, 21]. In an experimental ex vivo study by Battersby et al., double gloving was associated with a reduction in knot-tying quality. Thus, which risk is more serious for the patient, a qualitatively poorer knot or the risk of infection, must be considered [2]. Balancing the risks is important, especially as the loss of the protective function of the glove was proven to allow bacteria to penetrate through these lesions [17], and even small amounts of bacteria could be sufficient to trigger an implant or suture material-associated infection [31]. Despite surgical hand disinfection, germs can pass through corresponding glove punctures, even if the germ load at less than 100 CFUs was low [29]. The detection of S. hominis—a common commensal on human skin—in the BPTT represents a very likely scenario for contamination, as it could occur during surgery. The *B. cereus* found on the unprotected skin of surgeons is a spore-forming bacterium that may show pathogenicity in surgery [7] but is mainly thought to be responsible for acute diarrhoea in humans [25]. The spores are often not safely eliminated by common hand disinfectants and hand washing [30]. An undamaged glove could prevent infection. However, further studies should be performed on how long hand disinfection can keep the hands sterile during orthopaedic surgery.

Of note, mechanical stress loading and its damaging effects are rarely considered in the known standards for manufacturing and testing gloves of the European Committee for Standardization (CEN) European Standard (EN) EN455 or American Society for Testing and Materials (ASTM D3577). To test for damage, only a random water tightening test is mandatory. During this test, the gloves must be leak-tight for 2–3 min when filled with 1000 ml of water, and higher stresses are not tested [1, 13]. Thus, randomly tested, the glove and the entire batch are considered to be leak-proof and free from damage. According to the standard, the product batch is allowed an accepted quality level (AQL) of 0.65 (EN455); for example, for a batch size of 500,000 pieces, only 315 gloves must be randomly tested, among which 5 pieces may show damage (AQL 0.65; 5 damages per 315 gloves) and still allow the batch to be placed on the market [1, 13]. In the test of mechanical load, the unused glove must carry a weight of 100 N for a short time, and further tests, including a test of the glove after the mechanical load, are not intended [13]. Other infection protection products, such as condoms, are subject to higher quality (AQL of 0.25; 2 instances of damage per 315 condoms) and are tested and standardised by the industry in a more complex process than surgical gloves [6].

In this context, the integrity of the glove as protection for surgical staff must also be clearly mentioned. While the transmission of bacteria primarily represents a loss of asepsis and a risk of infection for the patient, microlesions play a major role in the transmission of pathogenic viruses to the surgical team. Studies with pass-through tests of viruses can be found in the literature, but most of them are 20 years old and older [5, 34]. At that time, a latex glove was considered virus-proof if it was sufficiently vulcanised, and a liquid must exist as a transport medium (sweat and bodily fluids) [19]. Given the size of viruses in the nanometre range, however, even the smallest lesions and longer operation times could be sufficient to achieve transmission. Burn et al. showed that epithelial cell particles of the surgeon could be detected in the suture material despite wearing a glove. It shows how vulnerable gloves are and how an exchange of biomaterial can occur [3]. Therefore, microlesions could represent a major health risk for the surgical team, especially for high-risk patients with viral infections such as hepatitis or human immunodeficiency virus (HIV) [8, 18]. The skin lesions on the fingers resulting from knot tying could possibly serve as a port of entry. The results of Giordano et al. could not be substantiated by our study. Giordano et al. reported no risk of perforation of surgical gloves during knot tying and suturing and stated that the skin abrasions on fingers that occurred must have been caused by friction; thus, there was no risk of perforation of surgical gloves [14]. However, the static setup of their study may have neglected soft-tissue-related bouncing effects that occur under real surgical conditions and produce saw-like movements that could have led to the lesions on the collected gloves. In a further experimental approach, arthroscopic suture materials for rotator cuff repairs were examined for their lesion-causing characteristics; here, the potential for glove damage was certainly evident [23]. Unfortunately, the study did not report data on the size of the lesions caused by suture material. Although Kaplan et al. confirmed the damage caused by gloves during arthroscopy, there was no indication of the size of the lesions in these relevant studies. Furthermore, as experimental studies do not reflect the exact surgical environment, the influences of movements, tissue elasticity, body fluids and mechanical loads are missing [14, 20, 23].

In this context, it must be clearly emphasized that an AQL of 0.65 (EN 455) with a production-related basic rate of lesions during glove production does not provide sufficient protection against viral infections. As limitation of the study, two arthroplasties (THA and TKA) and one arthroscopic procedure (RCR) were included. However, the influence of different knot tying techniques in various anatomical structures (e.g., tendon/fascia) was investigated. The knot tying process itself is subject to the individual preferences of each surgeon. Despite specific instruction on knot tying, it was difficult to standardize the procedure due to multiple factors (e.g., strength, direction and speed of pull). However, lesions occurred in the gloves of all participating surgeons, thus suggesting that the damage is not the result of individual preferences and practices. The subsequent analysis in the test laboratory did not allow us to distinguish between lesions caused by the production process or lesions occurring during the procedure. Here, an improved analysis of the lesions, e.g., digital or electron microscopic analysis, could provide more information. As only gloves with a positive water tightening test were examined under the microscope for damage, further damage could therefore be undetected. It cannot be excluded that smaller lesions that occurred intraoperatively were increased in size when removing the glove. The effects on the postoperative wound infection rate as well as possible infections of the medical staff were not investigated.

Based on the findings, more attention should be given to the intraoperative use of gloves. The glove is subject to wear from lesions during the procedures, which is intensified or increased by tying knots. In the standards, water-retaining gloves are considered to be tight and therefore impermeable to viruses and other pathogens. Microlesions destroy the protective barrier and thus protect the patient and the surgeon. Glove-changing algorithms and double gloving should be established in the daily routine to ensure the best possible protection. The clinical benefit of the study would be to increase the awareness of the surgical teams of intraoperative damage of surgical gloves, their main damage areas in the related surgeries and corresponding mindfulness for infection prevention for the patient and surgical team. Furthermore, knot tying should be recognized as the cause of microlesions, and intraoperative glove-changing algorithms should be established.

## Conclusion

The very process of surgical knot tying leads to microlesions and thus the loss of integrity of the glove. The protective function of the glove against the transmission of viral and bacterial pathogens could be lost due to the microlesions. In surgeons, the skin lesions resulting from knot tying could in turn represent a convenient port of entry for pathogens. Regular glove changes, especially after knot tying and at defined intervals, are recommended. Additionally, gloves should be subject to more stringent standards, as mechanical stress and protection of the surgical team and the patient are not sufficiently considered. A requirement that would emerge from this study could be that gloves should be optimized in design and material and tailored to surgical requirements to limit wear.

## Supplementary Information

Below is the link to the electronic supplementary material.

Supplementary file1 (PDF 79 KB)

## Abbreviations

AQL: Acceptable quality level
ASTM: American Society for Testing and Materials
BPTT: Bacterial pass-through test
CEN: European Committee for Standardization
CFU: Colony-forming unit
DAkkS: National accreditation organization of the Federal Republic of Germany
DIN: Deutsche Industrie Norm
EN: European Standard
HIV: Human immunodeficiency virus
IEC: International Electrotechnical Commission
ISO: International Organization for Standardization
N: Newton
PH: Pulling hand
RCR: Rotator cuff repair
SD: Standard deviation
LH: Looping hand
THA: Total hip arthroplasty
TKA: Total knee arthroplasty
