# Supplementary material for: Knot tying in arthroplasty and arthroscopy causes lesions to surgical gloves: a potential risk of infection
Source: Knee Surg Sports Traumatol Arthrosc. 2022 Sep 1;31(5):1824–32. doi: 10.1007/s00167-022-07136-7 (PMC10089991; doi:10.1007/s00167-022-07136-7)
Supplement: Supplementary file 1 — Supplementary file1 (PDF 79 KB) [file 167_2022_7136_MOESM1_ESM.pdf]

Editing Certificate

This document certifies that the manuscript

Knoting damages on surgical Gloves

prepared by the authors

Andreas Enz, Annett Klinder, Lucas Bisping, Christoph Lutter, Philipp Warnke, Thomas Tischer, Wolfram Mittelmeier, Robert Lenz

was edited for proper English language, grammar, punctuation, spelling, and overall style by one or more of the highly qualified native English speaking editors at AJE.

This certificate was issued on **August 16, 2022** and may be verified on the AJE website using the verification code **9EE2-3328-D258-D4D8-E9CE**.

Neither the research content nor the authors' intentions were altered in any way during the editing process. Documents receiving this certification should be English-ready for publication; however, the author has the ability to accept or reject our suggestions and changes. To verify the final AJE edited version, please visit our verification page at aje.com/certificate. If you have any questions or concerns about this edited document, please contact AJE at support@aje.com.

AJE provides a range of editing, translation, and manuscript services for researchers and publishers around the world. For more information about our company, services, and partner discounts, please visit aje.com.
